# Phenotypically distinguishable eosinophilic cells do not impact epithelial functions in a triple-culture *in vitro* intestinal model

**DOI:** 10.3389/fimmu.2025.1641651

**Published:** 2025-08-04

**Authors:** Christoph Benkstein, Laurin Mosig, Daniel Vondran, Heidi Schlichting, Lea Kissing, Bente Wohlert, Ida Gensmer, Larissa Nogueira de Almeida, Peter König, Kerstin Fibelkorn, Anna Kordowski, Stefanie Derer-Petersen, Christian Sina, Yves Laumonnier

**Affiliations:** ^1^ Institute of Nutritional Medicine, Hospital Schleswig-Holstein (UKSH), Lübeck, Germany; ^2^ Institute of Anatomy, University of Lübeck, Lübeck, Germany; ^3^ Airway Research Center North, Member of the German Center for Lung Research (DZL), Lübeck, Germany; ^4^ Fraunhofer Research Institution of Individualised and Cell-Based Medical Engineering (IMTE), Lübeck, Germany

**Keywords:** eosinophils, electron microscopy, triple culture system, Caco-2, HT29-MTX cells

## Abstract

The small intestine is a complex assembly of different cell types, such as enterocytes, secretory, immune, stromal and nervous cells. Due to this complexity, studying human tissue function *ex vivo* is challenging. As surrogate systems, *in vitro* co-culture models have been proven to be reliable and affordable. In this study, we used absorptive and secreting epithelial cell lines combined with differentiated eosinophilic cells to establish a triple-culture system to examine the impact of eosinophils on epithelial cell functions. We first differentiated an eosinophilic precursor cell line (EoL-1) using butyrate, forskolin, or dibutyryl-cAMP. In-depth characterization by real-time PCR, flow cytometry, functional assay and electron microscopy showed that only butyrate and dibutyryl-cAMP generated phenotypically distinct eosinophilic cells with different activation statuses, marked by differential expression of surface markers CD11c and CD62L, increased expression of eosinophil specific genes, and development of eosinophilic structural features. Then, a triple-culture system encompassing the enterocytic cell line Caco-2 and the secretory cell line HT29-MTX complemented with eosinophilic differentiated cells was established. Eosinophilic cells altered neither the proliferation nor survival of the culture. In order to get additional insights in possible changes of specific epithelial functions, we assessed the expression profile of different genes that are critical for various functions of the epithelia. The presence of eosinophilic cells did not affect the expression of crucial genes involved in intestinal barrier functions, nor did it modify the epithelial barrier function as demonstrated by electrical resistance and paracellular transport assays. However, mucus staining of the epithelial layer indicated that triple-culture with eosinophilic cells obtained using butyrate showed a tendency to a weaker mucus production. Furthermore, although the eosinophilic cells did not alter the epithelia, we observed the survival of butyrate-differentiated eosinophilic cells over a long period of time. Collectively, our data suggest that different triggers drive EoL-1 cells into phenotypically different eosinophilic cells with possibly distinct functions, mimicking the variability of eosinophils *in vivo*. Furthermore, this approach could be used as a stable triple-culture assay since differentiated eosinophilic cells showed no detrimental effect on epithelial functions.

## 1 Introduction

Although eosinophils were described more than a century ago, recent studies have shown that their functions extend far beyond their role as effector cells in the course of parasitic infection or in maladaptive type 2 inflammation. Recently, eosinophils have been recognized to play key functions not only in inflammatory response but also in various homeostatic and remodeling processes (1). Further, several studies in mice highlighted eosinophils to be more heterogeneous than initially thought (2, 3). In the murine lamina propria (LP) of the small intestine, two populations of eosinophils coexist (4), while in allergic inflammation, the eosinophils that home to the lung have a different phenotype, characterized by the expression of the integrin CD11c and co-stimulatory molecules (5, 6). Interestingly, although the gut is the organ with the highest number of eosinophils (7), their functions in gut homeostasis have been unclear for a long time. Recently, a study provided evidence that LP eosinophils of the small intestine support the development and barrier function of the gut epithelium (8). Further, transcriptomic analysis of murine eosinophils distinguishes active from basal populations of cells in the intestinal LP, based on the positive expression of CD80 and PD-L1 in active eosinophils, while these markers are not expressed in basal eosinophils (4). In addition to the LP eosinophils, another population of eosinophils located in the epithelial layer itself has been identified in murine small intestine (9). While this neglected population expresses a higher level of CD11c (10), their functions remain elusive. Although interactions between epithelium, myeloid and innate lymphoid cells in lungs have been well studied (11), there is still a gap in knowledge concerning the direct crosstalk between gut epithelium and eosinophils, even though the latter represent a large fraction of immune cells located in the small intestine.

In humans, functional studies typically use eosinophils isolated from the blood. However, they represent only a subset of eosinophils, the mature inactive ones, expressing CD11c, CD11b, CCR3 and Siglec 8, among others, but only low levels of activation markers compared to tissue eosinophils (12). As an alternative, the eosinophilic-like leukemia cell line (EoL-1) has long been described as a suitable source for generating eosinophils *in vitro* (13), with the use of different molecules to trigger EoL-1 differentiation. Butyrate has been widely used to differentiate EoL-1 into eosinophilic cells (14–16). Furthermore, forskolin (17) and dibutyryl (db)-cAMP (18–20) have also been used as alternatives to induce EoL-1 differentiation toward eosinophilic cells. As these studies employed various methods to characterize eosinophil phenotypes, there are inconsistent definitions of the differentiated cells. Consequently, it remains unclear whether these different approaches to differentiate EoL-1 are generating identical or distinct subsets of eosinophilic cells.

The Caco-2 and HT29-MTX human cell lines emulate the intestinal epithelial layer and provide an *in vitro* model for comprehending and investigating its functions. Caco-2 cells represent a model of small intestinal enterocytes (21–23). However, since Caco-2 cells do not secrete mucus (22), HT29-MTX has been introduced in a co-culture system to regain the mucosal function of the barrier (24), where their combination represents an easy, affordable and appropriate model for functional research as well as an ideal starting point for triple-culture systems to study the impact of immune cells (25).

Our study first aimed at characterizing the different eosinophilic cells obtained by differentiating EoL-1 using butyrate, forskolin, and db-cAMP at the transcriptional, protein and structural levels. We observed that butyrate and db-cAMP induced the expression of different eosinophilic markers at mRNA level, while forskolin showed little effect. However, while CD80 was expressed at low level in both, butyrate strongly up-regulated CD11c and CD62L, in contrast to db-cAMP-derived cells, suggesting functional differences between these two differentiated EoL cells. We then used these two differentiation models to assess the potential impact of different types of eosinophils on epithelial barrier functions. Interestingly, our triple-culture system encompassing EoL-derived cells and an epithelial co-culture of Caco-2/HT-29-MTX showed that eosinophilic cells, even with an active phenotype, did not alter the epithelial gene expression, proliferation, or functions, while a more tolerogenic type may regulate the production of mucus by the epithelium.

## 2 Material and methods

### 2.1 Culture and maintenance of cell lines

Caco-2 (ATCC #HTB-37) and HT29-MTX (ECACC #12040401) cell lines were obtained and kept in DMEM supplemented with L-glutamine (Gibco ThermoFisher Scientific), 1% fetal calf serum (FCS), 1% Penicillin-Streptomycin (P/S), and 1% sodium pyruvate (all from Life Technology) for Caco2 and DMEM with 10% FCS, 1% P/S and 1% non-essential amino acids (Life Technology) for HT29-MTX. EoL-1 cells (DSMZ #ACC386) were obtained from ATCC and kept in RPMI 1640 supplemented with L-glutamine (Gibco ThermoFisher Scientific), 10% FCS and 1% P/S. The cells were cultivated in 250 mL flasks with a growth surface of 75 cm^2^ (T75) or 50 mL flasks with 25 cm^2^ growth surface (T25) and kept in an incubator at 37°C with 5% CO_2_. To avoid confluency, cells were split every 3–4 days and seeded out at 0.5 x 10^6^ cells per mL. Cells were kept in culture until reaching passage 23.

### 2.2 Isolation of human blood eosinophils

To control the expression of key eosinophilic markers such as CCR3 or Siglec 8, human blood was harvested from human donors in presence of EDTA. Ethical clearance was granted by the ethics committee of the University of Lübeck (approval number: AZ 19-233). Voluntary and informed consent donors were informed about the aim and the procedure and signed a consent. Eosinophils from the blood were purified using Percoll gradient centrifugation, red blood cell lysis (26) followed by magnetic selection using an Eosinophils blood isolation kit according to manufacturer’s recommendations (Miltenyi).

### 2.3 Differentiation of EoL-1

To differentiate EoL-1 cells, 0.5 x 10^6^ cells per mL were seeded in RPMI-1640 supplemented with L-glutamine, 10% FCS, and 1% P/S and differentiated using either 250 μM butyrate (Millipore Merck), 50 μM forskolin (Enzo Life science), or 100 μM db-cAMP (STEMCell technology). After 3 days of incubation, part of the cells was harvested for further analysis or used to prepare triple-cultures with Caco-2 and HT-29-MTX cells.

### 2.4 Co- and triple-culture systems

Culture systems comprising Caco-2/HT29-MTX supplemented or not with EoL-1 or EoL-derived cells were established either in a plain 2D culture 6 well plate (Sarstedt) or in Transwell inserts (0.4 μm pore size, Corning) in DMEM with 10% FCS and 1% P/S and 1% non-essential amino acids. Briefly, Caco-2 and HT29-MTX cells were seeded at a ratio 9:1 (27) in absence or presence of EoL-1 or EoL-1-derived cells, at a ratio EoL 1:1,000 Caco-2/HT29-MTX. Cells were kept in culture up to 19 days, while culture medium was exchanged every second day.

### 2.5 Real-time proliferation analysis of triple-culture

The InCucyte SX5 system (Sartorius) was used as an automated analysis system for real-time and quantitative monitoring of cell cultures. The triple-culture of Caco-2, HT29-MTX and EoL-1-derived eosinophilic cells were seeded into a 24 well plate. Continuous growth of cultures was quantified every 4 hours for a total of 72 hours by using a 20x objective and the Adherent Cell-by-Cell scan type of the IncuCyte Imaging System (Essen BioScience). The average Phase Area Confluence (PAC) was normalized to the PAC at 0 days 0 hours 0 minutes, and the analysis was conducted using AI Confluence analysis software (Satorius).

### 2.6 Total RNA extraction and mRNA analysis

Total RNA was extracted using an innuPREP RNA Mini Kit 2.0 (Analytic Jena) according to manufacturer recommendation. Digestion of genomic DNA (gDNA) was performed during the extraction steps. Elution was performed using 40 μl RNase-free water. The concentration and the purity of the RNA was determined using a nano spectrophotometer (Nabi UV/Vis) and the quality using an Agilent RNA6000 Nano Chip Kit (Agilent Technologies). Synthesis of cDNA was performed using a RevertAid H Min.M-MuLV RevTrans reverse transcriptase (ThermoFisher Scientific) in presence of RNase inhibitor. Semi-quantitative PCR was performed using PerfeCTa SYBR Green SuperMix (Quantabio), according to manufacturer’s recommendations. The housekeeping gene *β-ACTIN* was used as a reference gene; *RNASE2, RNASE3, PGR2, PTAFR, TNFR1* and *TNFR2* were target genes for eosinophils, *KFL4*, *MUC5AC*, *Occludin*, *TJP-1, F11R*, *Claudin 2, CYP4A22, ANPEP, TRPV6, GATA4, ALPI, and HES1* were target genes to explore epithelial functions. Primer sequences are shown in **Table 1**. Target genes Ct were normalized to the Ct *β-ACTIN* to obtain a ΔCt, and the abundance of target gene compared to *β-ACTIN* levels was shown as 2^-(Delta;Ct)^.

**Table 1:** Primer list.

Gene	Direction	Sequence (5´to 3´)
*β-Actin*	for	ACA TCC GCA AAG ACC TGT ACG
	rev	TTG CTG ATC CAC ATC TGC TGG
*RNASE2*	for	CTC CCA GCA ATG CAC CAA TG
	rev	GGA GGG TCT CGT CGT TGA TC
*RNASE3*	for	GCC ATC CAG CAC ATC AGT CT
	rev	CCT GGT CTG TCT GCA TAC GT
*PGR2*	for	CTTCCACCTTTGAGACCCCT
	rev	GGATGCCCACCACTTTTACTG
*PTAFR*	for	GTCCTTGGTCATCTGGGTGG
	rev	GCGGAACTTCTTGGTGAGGA
*TNFR1*	for	TGCAGAGAGAAGCCAAGGTG
	rev	GGCTGGAATCTGTGTCTCCC
*TNFR2*	for	GACCTGTCCCTGAACCCTAT
	rev	CATTCCCACCTTTGTTGGA
*KFL4*	for	CCATCTTTCTCCACGTTCG
	rev	ATCGGATAGGTGAAGCTGCA
*MUC5AC*	for	CTGTGTCAAAGTGTGCCTGC
	rev	TTGATCACCACCACCGTCTG
*OCLN*	for	GCA TTG CCA TCT TTG CCT GT
	rev	TGA GCA GTT GGG TTC ACT CC
*TJP-1*	for	AGA CCT TGA CTC CAG ACA GC
	rev	GGT ACT TGC TCG TAA CTG CG
*F11R*	for	TAA CAT CCC CTC CTC TGC CA
	rev	TTC TCC TTC ACT TCG GGC AC
*CLDN2*	for	CTT TTG GGC ACA CTG GTT GC
	rev	TGT CTT TGG CTC GGG ATT CC
*ANPEP*	for	GGA CCA CCT GCA GGAGGC T
	rev	GTT CAT GAT GTC CCGCAC G
*TRPV6*	for	ACC TAT GCT GCC TTTGCC AT
	rev	GGG AGA TGA GAC CTCTGG GT
*HES1*	for	CTACCCCAGCCAGTGTCAAC
	rev	GGTCACCTCGTTCATGCAC
*ALPI*	for	CAATGTGGACAGACAGGTGC
	rev	TGACTTTCCTGCTTGCTTGG
*GATA4*	for	TCT ACA TGA AGC TCCACG GG
	rev	TAT TCA GGT TCT TGGGCT TCC

### 2.7 Flow cytometric analysis and antibodies

Phenotypic characterization of cells was performed on an ATTUNE NxT flow cytometer (ThermoFisher Scientific). Cell viability analysis was performed using 4′,6-diamidino-2-phenylindole (DAPI). Monoclonal phycoerythrin (PE)-labeled antibody (Ab) against CD11c (clone Bu-15), allophycocyanin (APC)-labeled Ab against CD80 (clone 16-10A1), PE-Cy7-labeled Ab against CD101 (clone Moushi101), Brilliant Violet (BV) 605-labeled Ab against CD62L (clone DREG-56), APC-labeled Ab against Siglec 8 (clone 7C9), fluorescein isothiocyanate (FITC)-labeled Ab against CD63 (clone H5C6), and PE-labeled Ab against CD193/CCR3 (clone J073E5) were purchased from eBioscience. Stainings were performed in PBS/BSA (0.5%) in presence of Fc receptor blocking reagent (TrueStain, Biolegend). Analysis of flow cytometry data were performed using FlowJo 10 (Becton Dickinson).

### 2.8 TNF-α stimulation

EoL-1 and EoL-derived cells were resuspended at 0.4 x 10^6/^mL and seeded for 30 minutes. Cells were stimulated with 100 ng/mL TNF-α (Peprotech) for one hour, harvested, blocked in PBS/BSA and stained for CD63 on their surface as marker of degranulation (28). Cells were then analyzed by flow cytometry.

### 2.9 Light and electron microscopy

For light microscopic examination, cells were cytospun using a Shandon Cytospin 2 (Thermo Fisher Scientific), fixed and stained using hematoxylin (Merck Millipore) and eosin (Carl Roth) (H&E) before microscopic examination. Further, H&E was used to stain Transwell cross-sections. Pictures were taken on a Zeiss microscope equipped with an Axiom 3 camera. For electron microscopy, EoL and EoL-derived cells (1 x 10^6^) were collected and fixed in 1 mL Monti Graziadei fixative. Similarly, a duodenal biopsy sample was fixed in paraformaldehyde before being transferred into Monti Graziadei fixative. The tissue was obtained using the ethical clearance granted by the ethics committee of the University of Lübeck (approval number: AZ 19-233). Voluntary and informed consent donor was informed about the aim and the procedure and signed a consent. Cells or tissues were then washed in 0.1 M Na- cacodylate buffer, stained with 2% osmium tetroxide, and washed again in 0.1 M Na-cacodylate buffer for 30 minutes. After gradual dehydration in ethanol, samples were placed in propylene oxid, and embedded in araldite for 2 days at 60°C. The resulting blocks were cut into 70–90 nm sections on an Ultracut E (Leica) and the sections got contrasted in an EM AC20 (Leica) with 0.5% uranyl acetate and 1% lead citrate. Pictures were taken on a JEM.1011 (Jeol) transmission electron microscope.

### 2.10 Trans epithelial electrical resistance measurements

Co-culture of Caco-2/HT29-MTX, with or without EoL-1 and EoL-1-derived eosinophils were cultivated up to 19 days on Transwell membranes in DMEM with 10% FCS and 1% P/S and 1% non-essential aminoacids. Before each medium change (every second day), TEER was measured for all samples using an EVOMx3 epithelial voltmeter equipped with the STX2-Plus Electrode World Precision Instruments). Raw resistance values were normalized against TEER across a blank membrane without cells, then corrected by the area of the insert, resulting in the TEER expressed as ohm x cm^2^.

### 2.11 FITC dextran measurements

Co-culture of Caco-2/HT29-MTX, with or without EoL-1 and EoL-1-derived eosinophils were cultivated for 10 days on Transwell membranes with changes in the medium every second day. At the end of the cultivation, medium was replaced by fresh PBS and 150 µl of 1 mg/mL Fluorescein Isothiocyanate (FITC)-Dextran (Sigma-Aldrich) was added to the apical Transwell chamber. After 20 minutes incubation in the dark at room temperature, the basolateral chamber medium was collected and the amount of FITC dextran measured at 485/535 nm, using a Spectra Max ID3 spectrophotometer (Molecular Devices) and normalized to a Transwell containing only medium.

### 2.12 Membrane cross-section analysis

Co-culture of Caco-2/HT29-MTX, with or without EoL-1 and EoL-1-derived eosinophils were cultivated for 10 days on Transwell membranes with changes of medium every second day. Membranes were then fixed in 4% paraformaldehyde/PBS, cut out from the insert, fixed in agarose (2% in bidistilled water) and embedded in paraffin. Blocks were cut to obtain cross sections. Membranes were then stained with H&E and Periodic acid Shiff (PAS) to assess the mucus production and pictures were taken using a Zeiss microscope equipped with an Axiom 3 camera. Equal lengths of cross-sections were evaluated for the area occupied by the epithelium using Image J software (NIH), normalized to their length to obtain the average height of the epithelium expressed in μm. Assessment of PAS staining was performed by visual examination of 3–5 areas of membrane’s sections after staining. For each independent experiment, the mean number of PAS positive granules was calculated.

### 2.13 Statistical analysis

Statistical analysis was performed using GraphPad Prism version 10 (GraphPad Software, Inc.). Experiments were performed as biological replicates, each data point being generated using different batch or passage of each cell lines. Normal distribution of data was tested using the Kolmogorov-Smirnov and D’Agostino-Pearson tests. When groups were normally distributed, statistical differences between two groups were analyzed by unpaired t-test. Comparisons involving multiple groups were first analyzed by ANOVA followed by Tukey’s test. When groups were not normally distributed, they were analyzed using Mann-Whitney U (two groups), or ANOVA on ranks (multiple groups) followed by a Dunn’s multiple comparison test. A p value < 0.05 was considered significant and represented as follows: *p < 0.05; **p < 0.01; ***p < 0.001.

## 3 Results

### 3.1 Butyrate and db-cAMP but not forskolin drive EoL-1 toward an eosinophilic phenotype

**Figure 1:**
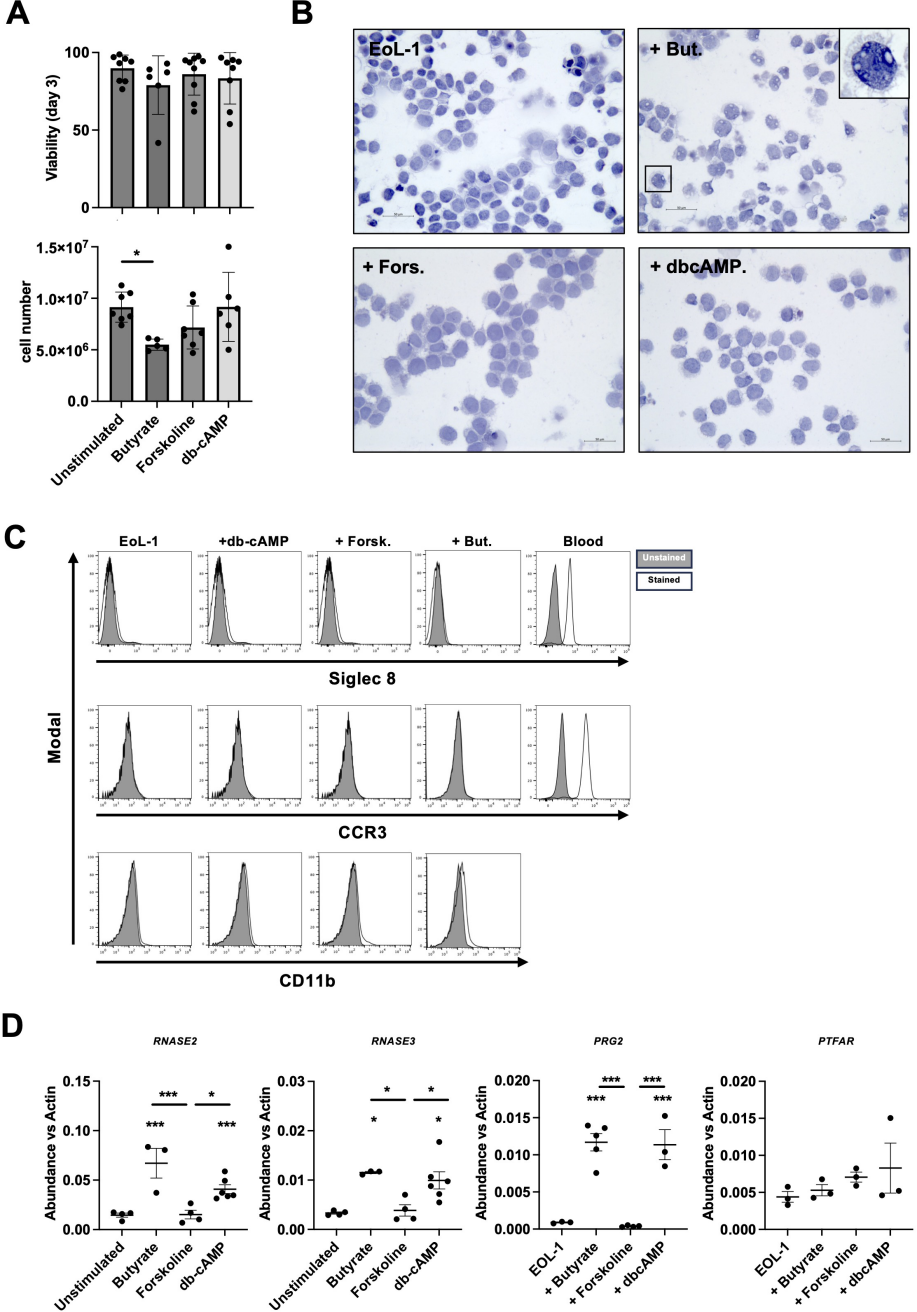
Characterization of the different EoL-1 cell populations activated by butyrate, forskolin and db-cAMP. Cells were cultured for 3 days with butyrate (+But.), forskolin (+Fors.) or db-cAMP (+dbcAMP). **(A)** Cell number was assessed by counting using a Neubauer chamber. Data show the mean value ± SEM of the number of living cells, n=6–7 independent stimulations. Statistical significance was tested by ANOVA followed by Tukey’s test. Asterisks show significance, *** p<0.001. Cell viability was tested by flow cytometry after DAPI staining. Data show the mean value ± SEM of the percentage of living cells, n=8–9 independent stimulations. Statistical significance was tested by ANOVA followed by Tukey’s test. Asterisks show significance, *** p<0.001. **(B)** Cell morphology was determined by visual examination of cells after cytospin. Pictures are representative of at least 3 independent stimulations. Bar represents 50 μm. In butyrate, higher magnification picture shows vacuoles. **(C)** Expression of eosinophilic markers. Data show the expression of Siglec 8, CCR3 (CD193) and CD11b at the surface of cells. Positiveness of Siglec 8 and CCR3 in blood eosinophils is shown as comparison. Histograms shown are representative of at least 3 independent experiments and show, for each marker, the unstained control (grey histogram) and the stained samples (black line). **(D)** mRNA analysis of eosinophil specific genes. Data show the mean value ± SEM of the abundance of *RNASE2, RNASE3, PRG2* and *PTAFR* mRNA vs *b-actin*, n=3–5 independent stimulations. Statistical significance was tested by ANOVA followed by Tukey’s test. Asterisks show significance, *p<0.05, ***p<0.001.

Blood eosinophils are a potent source of eosinophils but have poor survival capacity *in vitro* and do not have the same heterogeneous nature as tissue eosinophils (4). In contrast, the eosinophilic-like leukemia cell line (EoL-1) has been successfully used for many years to generate eosinophils using various triggers. We used 250 μM butyrate (29), 50 μM forskolin (17), or 100 μM db-cAMP (16) to differentiate EoL-1 and evaluated both cell viability and cell number, as indicator of cell proliferation, after three days of differentiation compared to unstimulated EoL-1. Viability assessment showed that bEoL, fEoL and dbcEoL exhibited a similar survival as EoL-1 over three days (**Figure 1A**). In contrast, cell counts showed that, while stimulation with forskolin (fEoL) and db-cAMP (dbcEoL) resulted in a similar number of cells compared to untreated EoL-1, butyrate treatment (bEoL) resulted in a significantly lower number of cells (**Figure 1A**), suggesting that these cells had a lower proliferative capacity and may have entered a strong differentiation program. Furthermore, H&E staining showed similar morphologies between EoL-1, fEoL, and dbcEoL, while bEoL exhibited vacuolar structures (**Figure 1B**), that have been associated with active inflammatory eosinophils (6, 30). To compare the differentiated EoL-1 cells to human native blood eosinophils, we performed a cell surface staining using classical eosinophilic surface markers. Intriguingly, neither butyrate, forskolin, nor db-cAMP triggered the expression of Siglec 8 and CCR3 (CD193) at the surface of EoL-1-derived cells, in marked contrast to blood eosinophils (**Figure 1C**). Cells were additionally stained for CD11b, a key adhesion molecule for eosinophils (31). Butyrate exposure drove the expression of CD11b at the surface of bEoL, although at a low level (**Figure 1C**), while fEoL and dbcEoL cells remained negative. To further characterize the phenotypes of the differentiated cells, we performed RNA analysis by semi-quantitative PCR. Our data showed that both butyrate and db-cAMP equally increased the mRNA expression of genes encoding eosinophilic specific molecules, such as *RNASE3*, *RNASE2*, and *PGR2* (**Figure 1D**), while forskolin had no effect. In contrast, no treatment induced the expression of *PTAFR*, encoding for the platelet activating factor receptor. Altogether, our data indicates that butyrate and db-cAMP are potent inducer of eosinophilic differentiation of EoL-1.

### 3.2 Butyrate and db-cAMP drive EoL-1 into different eosinophilic cells

**Figure 2:**
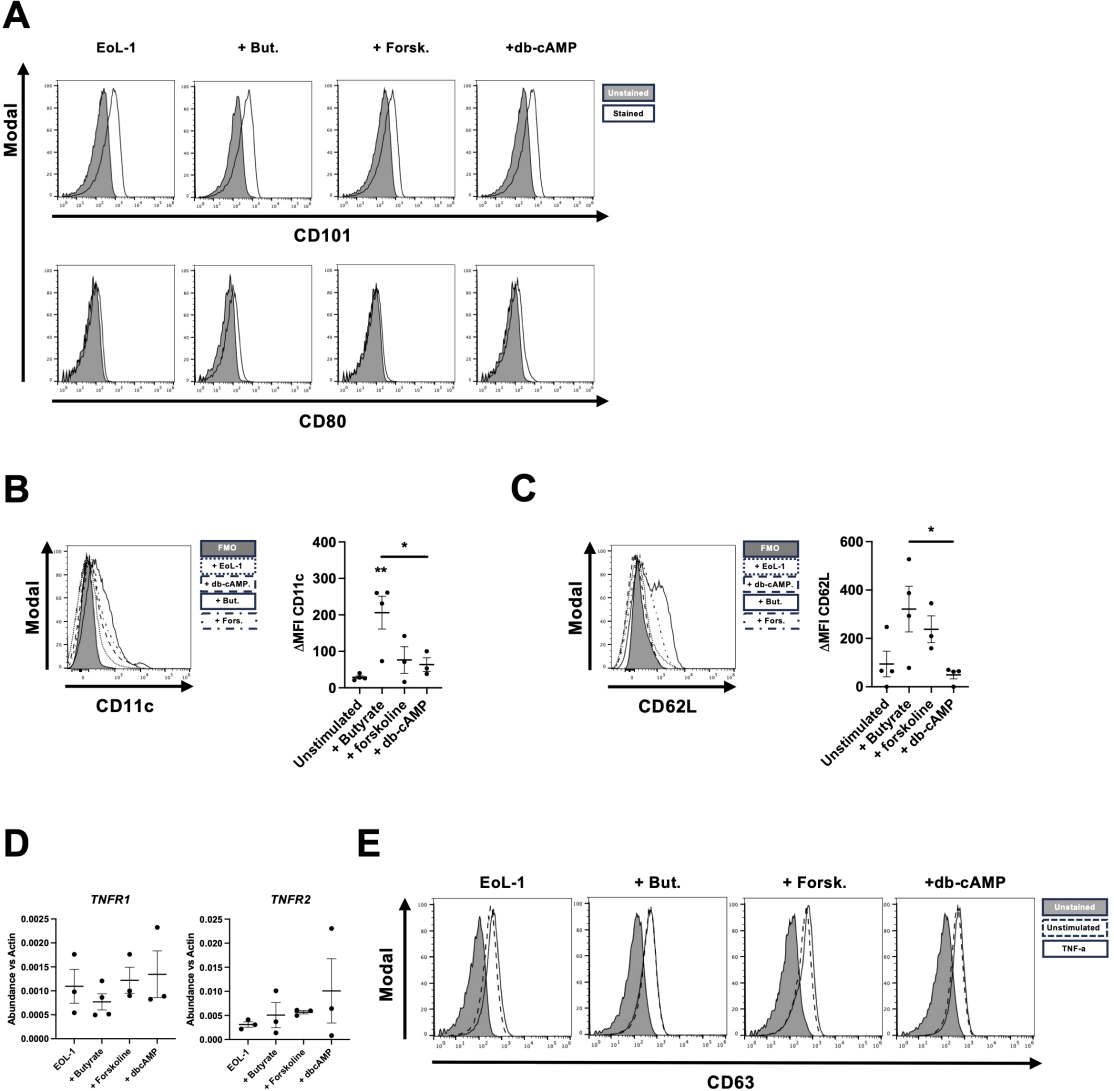
Butyrate triggers an strong eosinophil phenotype in EoL-1 cells. Activation of EoL-1 differentiated with buryrate, forskolin and db-cAMP was assessed **(A)** Expression of activation markers CD101, and CD80 was determined by flow cytometry. Data shown are representative of 2–3 independent experiments and show, for each marker, the unstained control (grey histogram) and the stained samples (black line). **(B)** Flow cytometric analysis of the expression of CD11c, at the surface of EoL-1 and EoL-derived cells. Histograms are representative of 3 independent stimulations and show unstained control (grey), unstimulated EoL-1 (dotted line), db-cAMP stimulated EoL-1 (dashed line), butyrate stimulated EoL-1 (black line), and forskolin (dot-dash mixed line). Scatter plot shows the mean fluorescence intensity of CD11c expression at the surface of EoL-1 and derived cells, normalized to the unstained control (DeltaMFI) ± SEM, n=3. Statistical analysis was performed using ANOVA followed by Tukey’s test. Asterisks show significance, *p<0.05 and **p<0.01. **(C)** Flow cytometric analysis of the expression of CD62L, at the surface of EoL-1 and EoL-derived cells. Histograms are representative of 3 independent stimulations and show unstained control (grey), unstimulated EoL-1 (dotted line), db-cAMP stimulated EoL-1 (dashed line), butyrate stimulated EoL-1 (black line), and forskolin (dot-dash mixed line). Scatter plot shows the mean fluorescence intensity of CD62L expression at the surface of EoL-1 and derived cells, normalized to the unstained control (DeltaMFI) ± SEM, n=3. Statistical analysis was performed using ANOVA followed by Tukey’s test. Asterisks show significance, *p<0.05. **(D)** mRNA analysis of TNF receptors expression. Data show the mean value ± SEM of the abundance of *TNFR1* and *TNFR2* mRNA vs *b-actin*, n=3–4 independent stimulations. **(E)** Flow cytometric analysis of the presence of CD63 at the surface of EoL-derived cells upon TNF-α stimulation. Histograms are representative of 3 independent stimulations and show unstained control (grey histogram), unstimulated EoL-1 (dashed line) and TNF-α stimulated EoL-1 (black line) for each condition.

Further, we investigated if our differentiated cells resemble more the active or basic subset of eosinophils (4, 6). The protein CD101, a marker of inflammatory eosinophils (6, 32), remained stable between the EoL-1 and the eosinophilic cells, while bEoL and dbcEoL expressed the co-stimulatory molecule CD80 at their surface compared to the EoL-1 and fEoL (**Figure 2A**). As CD80 is associated with inflammatory eosinophil phenotype, we assessed the expression of CD11c, which characterizes pro-inflammatory eosinophils in allergic asthma inflammation (5, 6, 32) and is expressed by intestinal tissue murine eosinophils (33). Only butyrate induced the expression of CD11c in differentiated EoL-1 (**Figure 2B**). Interestingly the L-selectin CD62L, associated with tolerogenic function of eosinophils (32) is also expressed at the surface of bEoL, at a significantly higher level than in dbcEoL (**Figure 2C**). Finally, although EoL-1 and EoL-derived cells alike expressed TNF-α receptors (TNFR)-1 and -2 at mRNA levels (**Figure 2D**), TNF-α did not trigger the translocation of CD63 (**Figure 2E**), an indicator of degranulation of eosinophils (28).

Altogether, our data suggests that butyrate was a potent inducer of EoL-1 differentiation toward a putative tolerogenic eosinophil phenotype, encompassing the expression of CD11c and CD62L. On the other hand, db-cAMP triggers the differentiation of a more active eosinophilic phenotype, as shown by the absence of CD62L, but the presence of CD80 expression.

### 3.3 Butyrate- and db-cAMP-derived EoL cells show ultrastructural features of eosinophils

**Figure 3:**
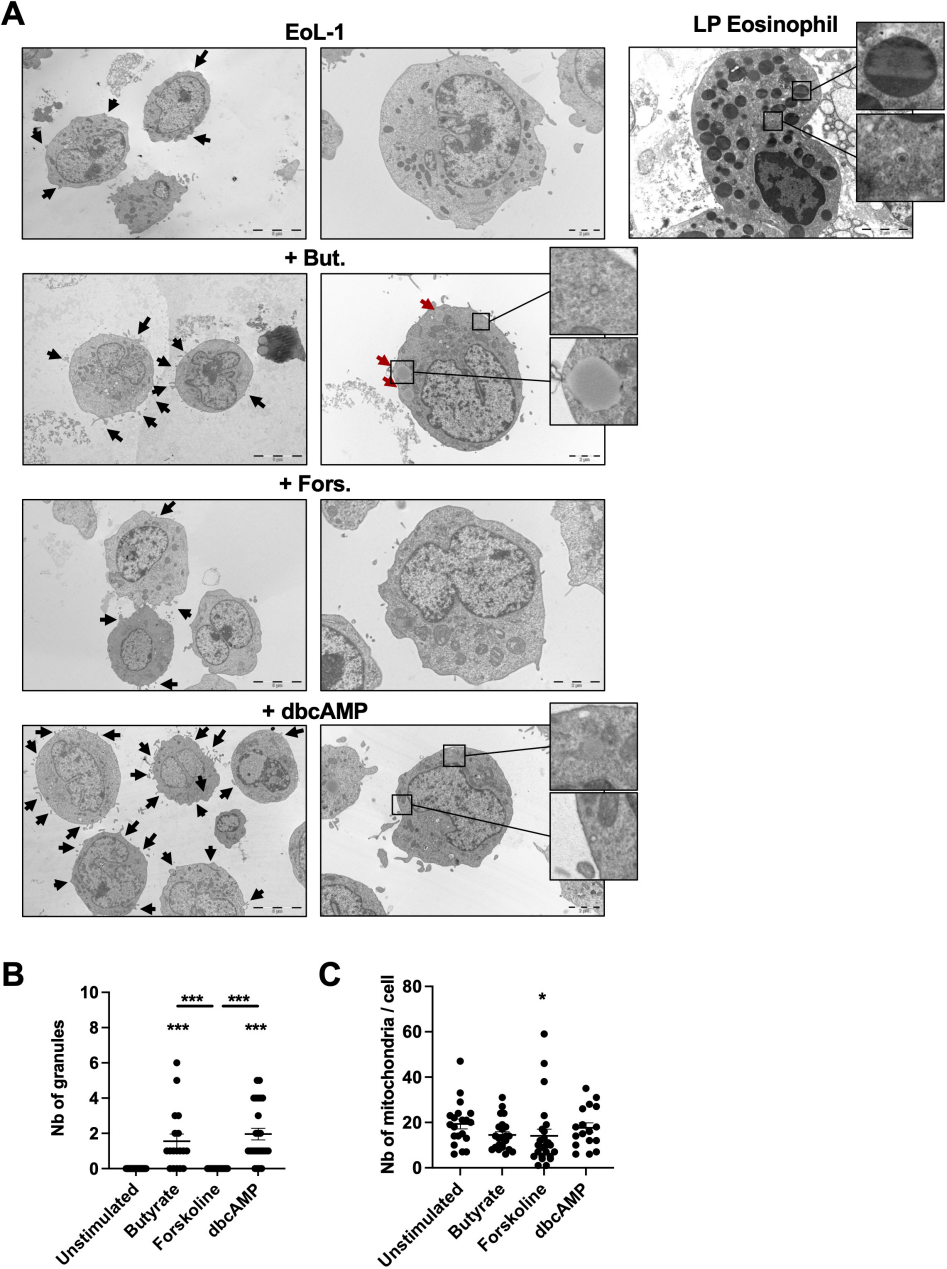
Ultra-structural characterization of EoL-1 and EoL-1-derived cell populations. **(A)** Cells were differentiated for 3 days with butyrate (+But.), forskolin (+Fors.) or db-cAMP (+dbcAMP), before fixation and preparation for electron microscopy. Pictures are representative of several cells examined. Left column, scale bars indicate 5 μm; right column, scale bars indicate 2 μm. Large granules are indicated with red arrows. Exemplary gut tissue eosinophil is shown as comparison. High magnification pictures show aspects of crystalline granule (upper) and sombrero vesicle (lower). On the right, tissue eosinophil from the duodenal lamina propria is shown as reference for eosinophilic morphology. **(B)** Number of granules per cell evaluated by electron microscopy examination. Data show the mean number of granules per cell ± SEM, n=3 independent stimulations. Statistical significance was tested by ANOVA followed by Tukey’s test. Asterisks show significance, ***p<0.001. **(C)** Number of mitochondria per cell evaluated by electron microscopy examination. Data show the mean number of mitochondria per cell ± SEM, n=3 independent stimulations. Statistical significance was tested by ANOVA followed by Tukey’s test. Asterisk shows significance, *p<0.05.

Eosinophils display very specific structural features, such as high numbers of granules exhibiting Major Basic Protein (MBP) crystals, but also other granular features such as sombrero vesicles (34), which is exemplified in the lamina propria eosinophil panel in **Figure 3A**. Intriguingly, although *PRG2*, *RNASE2*, and *RNASE3* mRNA could be detected in bEoL and dbcEoL, we could identify neither the crystalline forms of MBP, nor other granular features such as sombrero vesicles in bEoL and dbcEoL. However, we observed more pronounced signs of degranulation in these cells compared to undifferentiated EoL or fEoL (**Figure 3A**, black arrows). In agreement, both bEoL and dbcEoL presented structure similar to granules and on few occasions sombrero vesicles and showed a limited but significant increased number of granules compared to the EoL and fEoL cells (**Figure 3A** red arrows, **Figure 3B**). In contrast, the number of mitochondria in their cytoplasm was similar to what we observed in EoL-1 (**Figure 3C**).

Altogether, our data suggests that, although not as pronounced as in LP eosinophils, bEoL and dbcEoL displayed some features of classical eosinophils, in particular granules.

### 3.4 EoL-derived cells do not alter either the proliferation or the gene expression profile of epithelial cells in a triple-culture model

**Figure 4:**
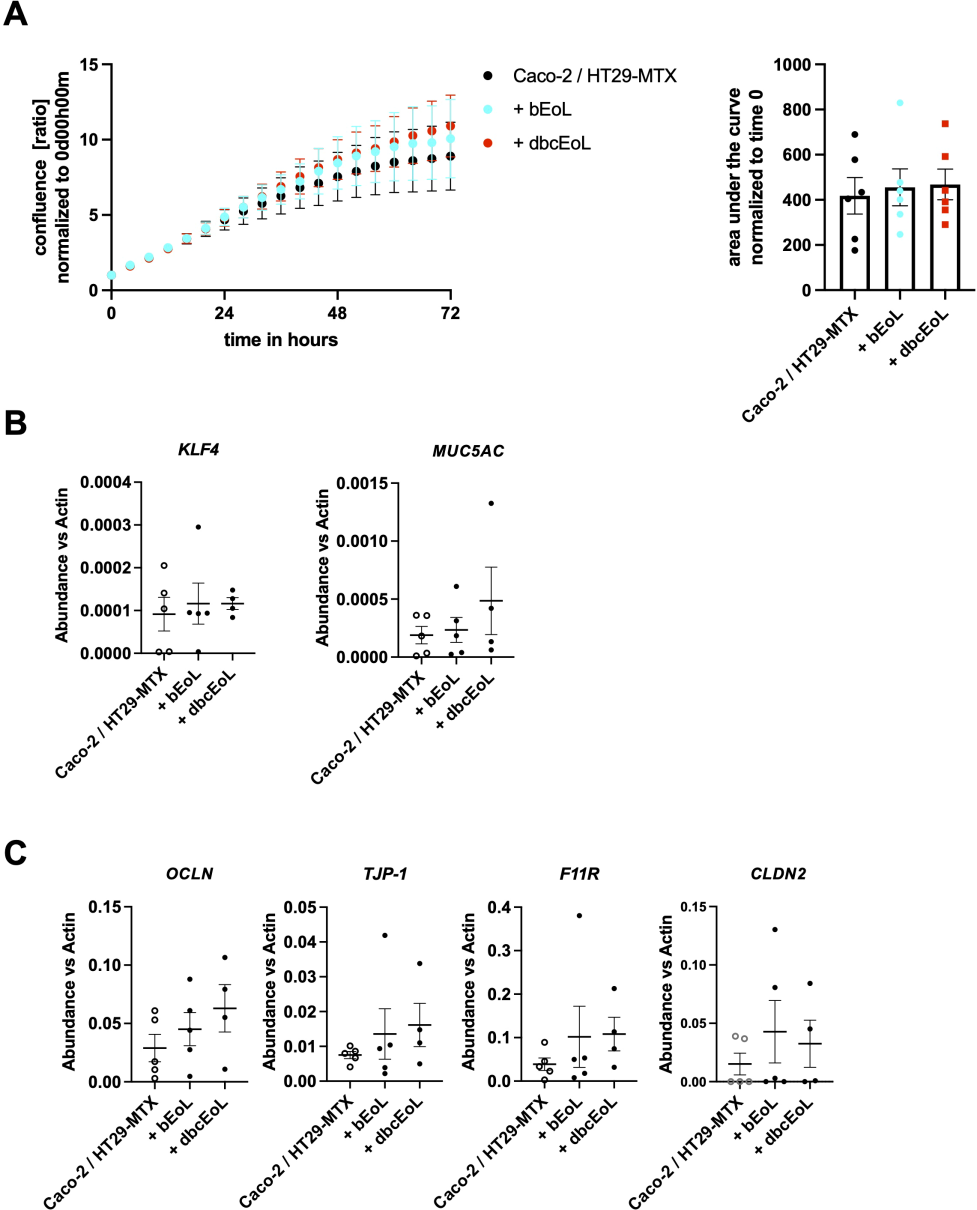
Co-culture of butyrate or db-cAMP EoL-derived cells with epithelial cells does not alter gene expression or proliferation of epithelial cells. EoL-derived cells generated using butyrate (bEoL) or db-cAMP (dbcEoL) were cultivated together with Caco-2/HT29-MTX epithelial cell lines. **(A)** Proliferation of co-culture in absence or presence of bEoL or dbcEoL was monitored for 72 hours using a live cell image system (InCucyte), which measures cell density every 4 hours. Data show the confluence normalized to the original seeding of the cells (0 days 0 hours 0 minutes) expressed as a ratio. Scatter plot shows the mean value of the area under the curves of the different culture conditions ± SEM, n=5–6 independent experiments. **(B)** After 8 days of culture, total RNA were isolated and expression of genes involved in epithelial function (*KLF4*, *MUC5AC*) or **(C)** barrier functions (*OCLN, TJP-1, F11R*, and *CLDN2*) was assessed by semi-quantitative real-time PCR. Scatter plots show the mean abundance of the gene compared to *b-actin* ± SEM, n=3–5 independent experiments.

As eosinophils are present not only in the lamina propria but also in the epithelium itself (33), we aimed at investigating the impact of CD11c^+^ and CD11c^-^ eosinophilic cells on epithelial homeostasis, barrier function, and paracellular transport. Therefore, we established an *in vitro* triple-culture system encompassing both Caco-2 and HT-29-MTX cells at a ratio of 9:1 complemented with EoL-1, CD11c^+^CD62L^+^ bEoL or CD11c^-^CD62L^-^ dbcEoL at a ratio of 1000:1 for 3, 8, or 10 days. We first analyzed the proliferation of the cells Caco-2/HT29-MTX complemented with bEoL or dbcEoL, using Incucyte. In none of the conditions we could observe an impact on the proliferation rate of the Caco-2 and HT-29-MTX coculture when complemented with bEoL, or dbcEoL (**Figure 4A**). Further, after 8 days of cultures, mRNA analysis revealed that the presence of bEoL-1 or dbcEoL did not change the expression of genes such as *KLF4*, and *MUC5AC* involved in differentiation of enterocytes and goblet cells functions respectively (**Figure 4B**). Similarly, bEoL, or dbcEoL did not alter the levels of mRNA encoding for adhesion molecules involved in maintenance of epithelial integrity such as *OCLN* encoding for the tight junction protein Occludin*, F11R*, encoding for the Junction Adhesion Molecule (JAM)-A*, TJP-1* encoding for the Zonulin (ZO)-1 protein, and *CLDN2* encoding for Claudin 2 (**Figure 4C**).

### 3.5 Butyrate and db-cAMP-derived EoL cells do not alter the barrier functions of the epithelium

**Figure 5:**
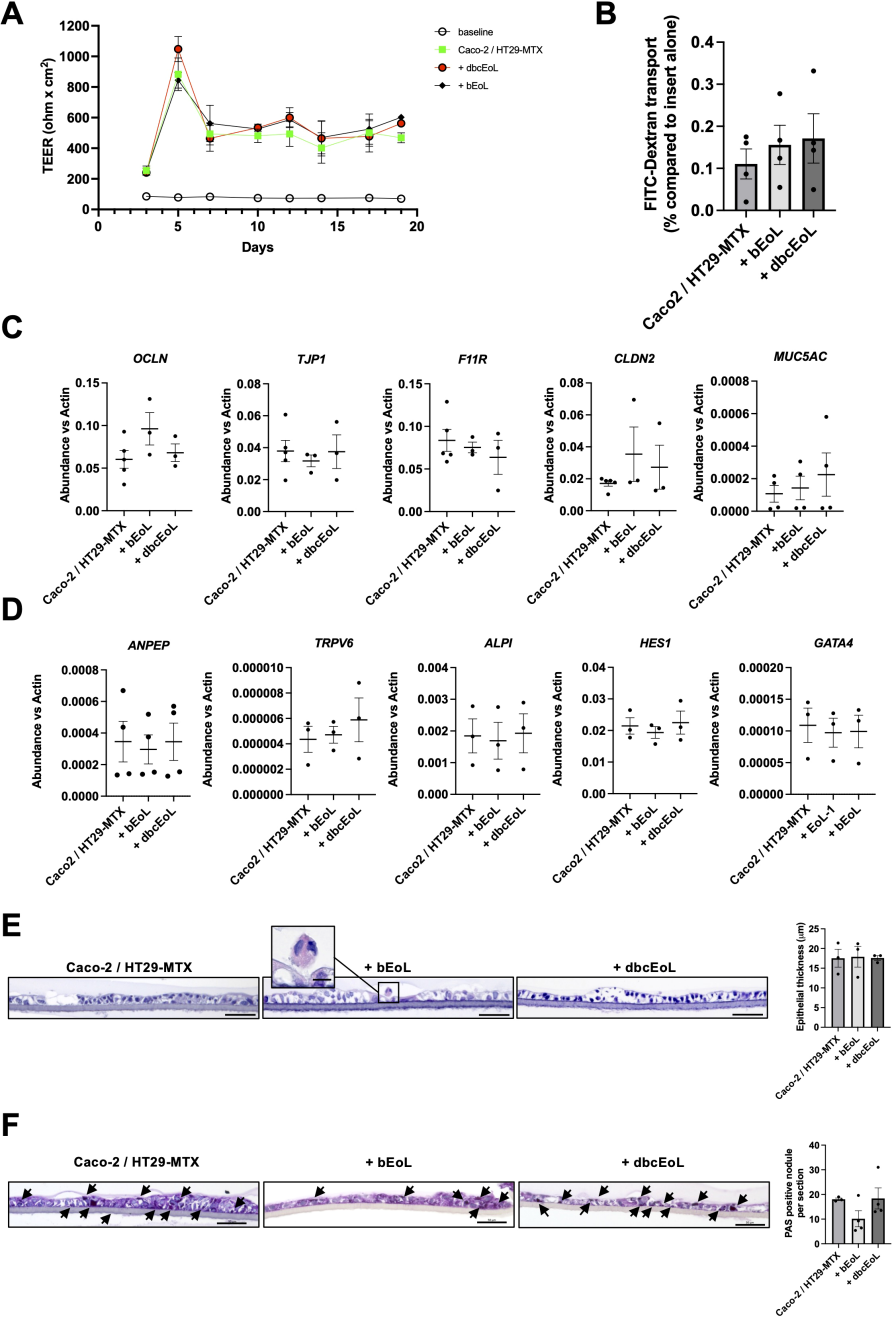
Triple-culture including EoL-derived cells do not alter the barrier function of an *in vitro* model of epithelium. EoL-derived cells generated using butyrate (bEoL) or db-cAMP (dbcEoL) were cultivated together with Caco-2/HT29-MTX epithelial cell lines in a Transwell system. **(A)** Transepithelial electrical resistance (TEER) was measured every second day after changes of the medium. Data show the mean value of the TEER (in ohm x cm^2^) of the different culture conditions ± SEM, n=7–8 independent experiments. Open circles show the baseline of the membrane without any cells. **(B)** FITC-dextran transfer from the upper to the lower compartment of a Transwell using different culture conditions at day 10. Data show the percentage of FITC fluorescence transport compared to the passive transport of an insert alone (100%, not shown) ± SEM, n=3-4. **(C)** Expression of genes involved in barrier function at day 10 assessed by semi-quantitative real-time PCR. Scatter plots show the mean abundance of the genes compared to *b-actin* ± SEM, n=3–5 independent experiments. **(D)** Expression of genes enriched in enterocytes (*ANPEP, ALPI*, and *HES1*), in proximal duodenal epithelial cells (*TRPV6*), and involved in epithelial differentiation and morphogenesis (*GATA4*) at day 10, assessed by semi-quantitative real-time PCR. Scatter plots show the mean abundance of the genes compared to *b-actin* ± SEM, n=4 independent experiments. **(E)** Thickness of the epithelial barrier in the different culture conditions. At day 10, after TEER and FITC-dextran measurements, Transwell membranes were harvested, embedded in paraffin and cut before staining with H&E and examined by light microscopy. Pictures of membrane cross-sections were taken, and equal lengths of membrane were analyzed for the area occupied by the epithelium by using ImageJ; scale bar indicates 50 μm. Scatter plot shows the mean thickness of the epithelium ± SEM, n=3 independent experiments. Insert in the bEoL panel documents the presence of an eosinophil, based on morphology and positive staining with eosin (scale bar indicates 10 μm). **(F)** Mucus production assessment. At day 10, after TEER and FITC-dextran measurements, Transwell membranes were harvested, embedded in paraffin and cut before staining with Periodic Acid Schiff (PAS) and examined by light microscopy. Pictures of membrane cross-sections were taken; scale bar indicates 50 μm. Assessment of PAS staining was performed by visual examination of 3–5 areas of membrane’s sections after staining. For each independent experiment, the mean number of PAS positive granules was calculated. Scatter plot shows the mean number of PAS positive granules, n=3–4 independent experiments.

To study possible changes in barrier integrity due to CD11c^+^CD62L^+^ or CD11c^-^CD62L^-^ eosinophils, we established a triple-culture epithelial barrier model seeded into a Transwell insert was set up. The Caco-2/HT29-MTX cells were able to mount an efficient epithelial barrier as shown by i) an increased and stable transepithelial electric resistance (TEER) already 7 days and up to 19 days after seeding (**Figure 5A**) and ii) the limited passive transfer of FITC-dextran, as marker for paracellular permeability and leakiness of the epithelial barrier from the upper to the lower compartments of the chamber (0.115 ± 0.035% of the amount of fluorescence transferred in absence of cells seeded on the membrane, **Figure 5B**, first bar). When Caco-2/HT29-MTX were supplemented with bEoL, or dbcEoL, the TEER was not altered from day 7 till day 19 compared to Caco-2/HT29-MTX alone (**Figure 5A**). Similarly, the evaluation with FITC-dextran transport at day 10 revealed a similar translocation of the labeled molecules in triple-culture conditions including bEoL, or dbcEoL compared to Caco-2/HT-29-MTX epithelial culture alone (**Figure 5B**). Furthermore, addition of EoL-1 derived cells to the co-culture did not alter the expression of genes encoding for proteins involved in barrier functions at day 10 (**Figure 5C**), or in enterocyte (*ANPEP, TRPV6, ALPI, and HES1*), and epithelial differentiation and morphology (*GATA4*) (**Figure 5D**). This expression could be directly attributed to the epithelial cells since neither bEoL nor dbcEoL express epithelial markers, except *F11R* (**Supplementary Figure 1**). Finally, to assess potential morphological changes in the epithelial layer in presence of EoL-1 derived cells, the inserts were embedded in agarose/paraffin, cut perpendicularly and stained with H&E. Microscopic examination showed a similar structural complexity of the epithelium in presence or absence of eosinophilic cells compared to epithelium alone. Furthermore, the thickness of the epithelial barrier was also similar in all conditions (**Figure 5E**). However, and although the expression of *MUC5AC* was not altered in triple-cultures compared to Caco-2/HT29-MTX, examination of cross-section after mucus staining with PAS showed a stronger staining in co-cultures and triple-culture with dbcEoL compared to bEoL (**Figure 5F**). Interestingly, bEoL-containing triple-cultures also showed persistence of eosin^+^ eosinophilic cell at day 10, suggesting that epithelial cells supported the survival of bEoL over a lengthy period of time.

## 4 Discussion

Although present in both the lamina propria and the epithelium, functions of eosinophils in the gastrointestinal wall remain elusive. Despite recent studies suggesting homeostatic functions for eosinophils in epithelial biology, clear evidence of direct interactions between eosinophils and epithelial cells remains scarce. To investigate this crosstalk, we established an *in vitro* triple-culture system by adding a long-standing eosinophilic cell line or its derivates (13, 35) to a well-established Caco-2/HT29-MTX *in vitro* co-culture model, which is commonly used to mimic epithelial functions (27). Here, we reported that butyrate and db-cAMP, but not forskolin, drove the differentiation of EoL-1 differently, leading to CD11c^+^ and CD11c^-^ eosinophilic cells, respectively. Furthermore, we observed in our triple-culture model that even when eosinophilic cells showed a pro-inflammatory profile, characterized by the expression of CD11c, they did alter neither enterocyte or mucosal cell gene expression and proliferation nor the barrier functions of the epithelium.

Butyrate has long been used to drive EoL-1 differentiation toward an eosinophilic phenotype (16, 20). Butyrate inhibits HDAC, yielding a continuous acetylation of histones H4 and H3 and resulting in cell differentiation, proliferation, and apoptosis (29, 36). Our data showed that butyrate increased the expression of mRNA encoding for eosinophil specific proteins, and costimulatory molecule CD80, as previously shown in pro-inflammatory lung eosinophils (6) and active gut eosinophils (4, 6). Similarly, the expression of CD11c was increased in bEoL. Although it is considered a pro-inflammatory marker in allergic lung murine eosinophils (6), CD11c expression is constitutive in healthy human blood eosinophils in contrast to mice (37), suggesting different functions of this integrin in human and murine systems. In contrast, bEoL also expressed CD62L, identified as a marker for lung tolerogenic resident eosinophils (32). Alternative to butyrate, db-cAMP has been used to activate astrocytes (38) and neurohybrid cell lines (39), and db-cAMP was reported to increase the expression of ECP in EoL-1 that then displayed a cytokine profile associated to eosinophils (20) and trigger the expression of *PRG2, EPX, CCR3*, and *IL5Ra* (40). In line with these findings, we observed an increased expression of *PRG2, RNASE2* and *RNASE3*, in a similar range to butyrate. However, neither CD11c nor CD62L expression were observed at the surface of dbcEoL, suggesting a more pronounced pro-inflammatory phenotype. Intriguingly, forskolin was unable to trigger an eosinophilic mRNA profile or structural changes, contrary to its previously reported positive effects on granules formation (17). However, even though butyrate and db-cAMP drove the differentiation of EoL-1, both eosinophilic cells lacked the expression of classical eosinophilic markers, such as Siglec 8. Further, in contrast to previously published data (41), bEoL and dbcEoL failed to express CCR3 on their surface. In addition, bEoL and dbcEol failed to mount a robust granular phenotype, as there was an absence of lucent crystalloids and limited evidence for the presence of sombrero vesicles, both structures being specific for eosinophils granules (42, 43). Intriguingly, the number of mitochondria was not affected, although a decrease of their number, together with changes in metabolic activities, correlate with eosinophil differentiation from precursors to immature and active eosinophils (44, 45). Lastly, bEoL and dbcEoL responded poorly to TNF-α stimulation, although expressing *TNFR1* and *TNFR2*, as they failed to translocate CD63 to the surface of cells as an indicator of degranulation of primary eosinophils (28). Altogether, the phenotype of bEoL mildly resembles human tolerogenic eosinophils, like the cells found in tissues (4, 32), while the dbcEoL resemble more active eosinophils, in particular due to the absence of CD62L and the expression of CD80.

Although it is well accepted that intraepithelial leukocytes (IELs) comprise up to 10% of the epithelial population, little is known about their impact on epithelial homeostasis and barrier functions. While the Caco-2/HT29-MTX co-culture system has been extensively used for drug discovery and studying the effects of food nutrients on the epithelium (25), it has been further refined as the basis for a triple-culture system. Indeed, a triple-culture system encompassing Caco-2/HT29-MTX and the Raji B cells has been used to study the permeability of the Caco-2/HT29-MTX barrier (46), and Raji B cells have been shown to improve tight junction integrity (47) and formation of specialized M cells (48). In contrast, THP-1 as a third component has been used to study inflammatory effects of nutrient activation macrophage-like cells upon barrier function (49, 50). Further, THP-1 and primary macrophages mimic a “leaky gut” phenotype when used in combination with Caco-2/HT29-MTX (51). Notably, these triple-culture systems have used THP-1 either seeded at the bottom of the lower chamber, or at the basolateral side of the membrane, i.e. not directly in contact with the epithelial barrier. In contrast, addition of active bEoL or dbcEoL to the Caco-2/HT29-MTX barrier did not alter the proliferation of the epithelium, nor did it affect its electrical resistance and paracellular transport, as shown by TEER and FITC-dextran assays. In line, mRNA analysis from triple-cultures showed no change in the expression of mRNA encoding adhesion molecules, such as *Occludin*, *F11R* (encoding for the junctional adhesion molecule (JAM)-A), *TJP-1* (encoding for zonulin) or *CLDN2 (*encoding for Claudin-2). Notably, Occludin, which provides stability to the epithelial tissue by creating a polarized barrier that supports homeostatic maintenance (52), is downregulated by MBP but not EDN secreted from AML14.3D10 eosinophils in an *in vitro* co-culture system with T84 epithelial colonic cells (53). Such reduction of *Occludin* results in increased permeability of the barrier, which might contribute to the development of conditions such as Crohn’s disease (54). The absence of such an effect in our model might be due to the absence of MBP crystals in bEoL and dbcEoL. Nevertheless, the apparent lack of effect of EoL-derived cells on epithelial barrier aligns with the absence of impact on barrier proteins and functions of eosinophil deficiency in mouse (8). This effect could not be attributed to a compensatory effect of the eosinophilic cells themselves, as they did express neither epithelial markers nor adhesion molecules but *F11R*. *F11R* encodes JAM-A and has not been formerly shown to be expressed by eosinophils, but is expressed by granulocytic neutrophils where it contributes to their capabilities to egress from the blood (55). Of note, although absence of eosinophils in a deficient Δdbl-GATA-1 mouse model results in a decrease in *Cyp4a12* (8), we could not explore that avenue, because of the absence of expression of its human homolog *CYP4A22* in the co-culture. Although Caco-2 cells are used to represent enterocytes, they do not always accurately replicate the duodenal phenotype of these cells, as they are derived from colonic tissue, which may explain the absence of *CYP4A22* expression. We also report no changes in expression of enterocytic specific gene such as *ANPEP*, *TRPV6, ALPI, HES1* and *GATA4*, encoding for the alanine-aminopeptidase (56), the calcium receptor TRPV6 (57), the transcription factor Hes-1 (Hairy and enhancer of split 1) (58), the intestinal alkaline phosphatase (59) and the GATA4 transcription factor (60), respectively, confirming the absence of detrimental effect of eosinophilic cells on enterocyte functions, epithelial differentiation, and morphogenesis. However, although the expression of mucosal genes such as *MUC5AC* or *MUC2* was not altered, bEoL triple-culture tended to show a lower number of PAS stained granules, suggesting that bEoL may regulate the mucus production at translational level. Interestingly, while activated eosinophils sustain increased mucus secretion (61, 62) or the increase of number of goblet cells (3), bEoL eosinophilic cells, resembling tolerogenic eosinophils by means of CD62L expression, may act to limit the mucus secretion.

Finally, although very rare to observe, and difficult to quantify, bEoL were still present 10 days after seeding, suggesting that the epithelium may support eosinophilic survival, similar to the 2 to 8 days of eosinophil survival reported in tissues (63). In the lung, epithelial cells promote the survival of eosinophils through secretion of neurotrophin (64). Further, IL-33, which is expressed by Caco-2, may be instrumental, as not only IL-33 released by duodenal epithelium is important for the eosinophil’s function in gut homeostasis (8), but also contributes to eosinophils survival (65).

Altogether, our data suggest that butyrate and db-cAMP drive eosinophilic differentiation of EoL-1 cells in distinct ways. Additionally, although we observed no clear influence of differentiated eosinophils on epithelial cell functions, we demonstrated that the triple-culture system is stable and contributes to the maintenance of eosinophilic cells. This could be an ideal and affordable model to further investigate the interactions between eosinophils and the intestinal epithelia and explore the role of eosinophils in nutrient activation of the epithelial barrier.

## Data Availability

The original contributions presented in the study are included in the article/**Supplementary Material**. Further inquiries can be directed to the corresponding author.

## Supplementary material

Supplementary Figure 1 bEoL and dbcEoL express F11R. EoL-derived cells generated using butyrate (bEoL) or db-cAMP (dbcEoL) were analyzed for *F11R* gene expression. Data show the mean abundance of *F11R* compared to *b-ACTIN* ± SEM, n=4 independent experiments.
