# Supplementary figures and images for: Phenotypically distinguishable eosinophilic cells do not impact epithelial functions in a triple-culture in vitro intestinal model
Source: Front Immunol. 2025 Aug 4;16:1641651. doi: 10.3389/fimmu.2025.1641651 (PMC12358489; doi:10.3389/fimmu.2025.1641651)

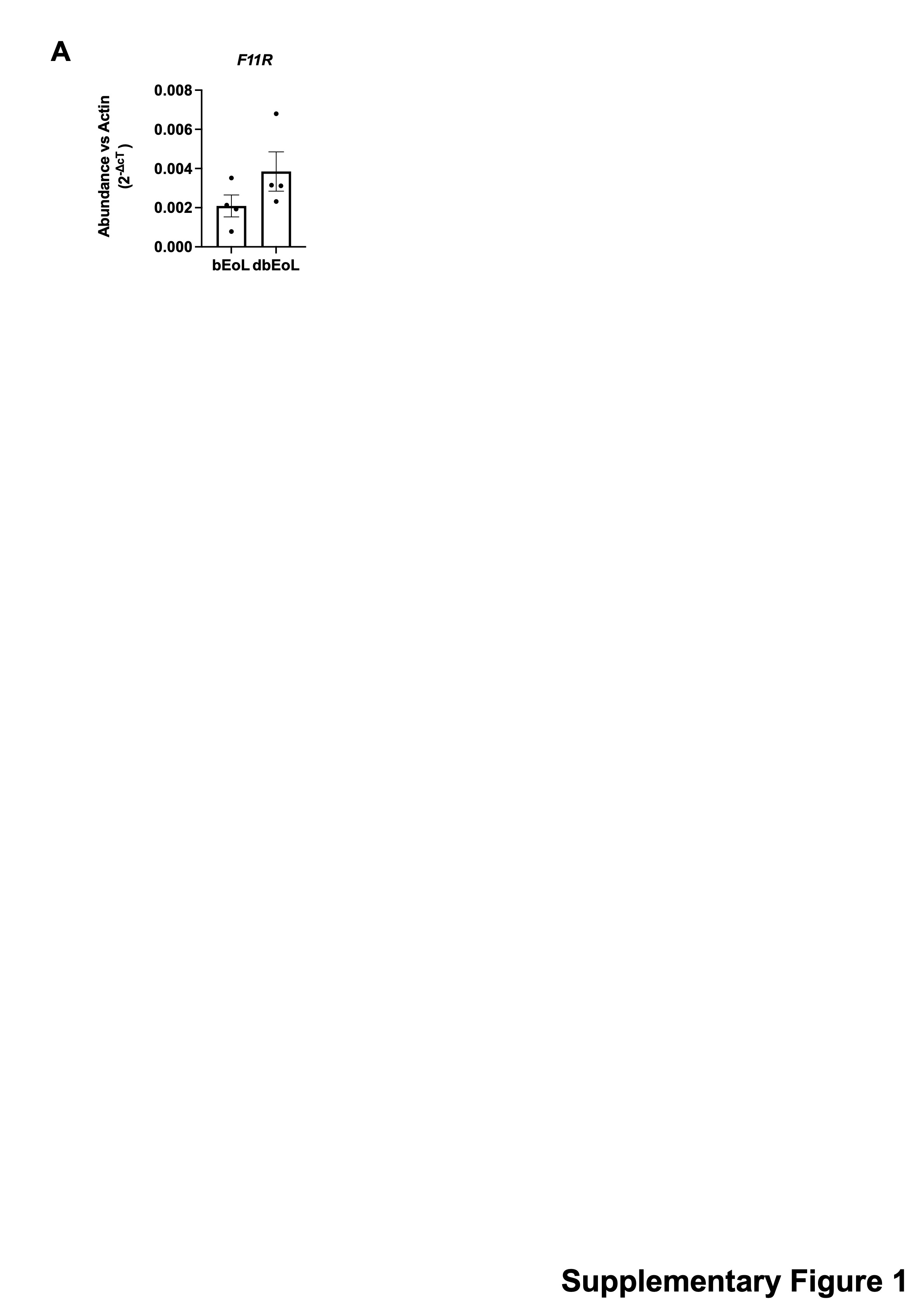

Supplement: Supplementary Figure 1 — bEoL and dbcEoL express F11R. EoL-derived cells generated using butyrate (bEoL) or db-cAMP (dbcEoL) were analyzed for F11R gene expression. Data show the mean abundance of F11R compared to b-ACTIN ± SEM, n=4 independent experiments. [file Image1.jpeg]
